# Post-traumatic stress disorder among Iranian women with genital mutilation: a cross-sectional study

**DOI:** 10.1186/s12978-022-01561-0

**Published:** 2023-04-12

**Authors:** Mahsa Abdollahzadeh, Roghaiyeh Nourizadeh, Niloufar Sattarzadeh Jahdi

**Affiliations:** 1 Student Research Committee, Tabriz University of Medical Sciences, Tabriz, Iran; 2 Midwifery Department, Faculty of Nursing and Midwifery, Tabriz University of Medical Sciences, Tabriz, Iran

**Keywords:** Female genital mutilation/cutting, Mental health, Post-traumatic stress disorder

## Abstract

**Background:**

The conflicting evidence on the relationship between female genital mutilation/ cutting (FGM/C) and post-traumatic stress disorder (PTSD) may be due to the differences in the prevalence and type of FGM/C in different societies. The present study aimed to assess the prevalence and severity of PTSD and its associated factors among Iranian women with genital mutilation.

**Methods:**

This cross-sectional study was performed on 155 women with genital mutilation aged 18–45 years referred to the health centers in Mahabad, located in west of Iran from October 2020 to April 2021. The participants were selected using convenience sampling method. After obtaining the informed consent form from the participants, the first researcher in the presence of a gynecologist determined the type of genital mutilation through the gynecological examination. The data were collected using demographic and obstetric characteristics and post-traumatic stress disorder checklist (PCL5) and analyzed using SPSS_21_ software. Further, independent t-test, ANOVA, Pearson correlation coefficient, and multivariate linear regression were used.

**Result:**

All 155 women (100%) had type 1 genital mutilation. Six women (3.9%) had PTSD. The mean (SD) score of the PTSD symptoms among the women was 27.73 (6.79) in the attainable score of 0–80. The age at FGM/C, level of education, and type of residence were considered as the predictors of the severity of the symptoms of PTSD, as explaining 48.1% of the variance.

**Conclusion and recommendation:**

In the present study, the prevalence and severity of PTSD among the participants were relatively low, which may be due to convenience sampling method used in the study, the limited injury in genitalia, and the social acceptance of the practice. The results indicated that the severity of the PTSD symptoms enhanced by increasing age at FGM/C and decreasing socio-economic levels. It is recommended to conduct the similar studies among women with other types of FGM/C.

## Plain language summary

Female Genital Mutilation/cutting (FGM/C) is prevalent in the south and west regions of Iran. Raising awareness on the consequences of FGM/C is essential for its prevention. In some societies, FGM/C triggers post-traumatic stress disorder (PTSD), which is a disorder resulting from an abnormal response to a traumatic event. It is the first research in Iran on PTSD following FGM/C. Our study was conducted among 155 women with genital mutilation referred to the health centers in Mahabad, located in west of Iran from October 2020 to April 2021. The participants were selected using convenience sampling method. The participants were examined for type of genital mutilation and asked to fill post-traumatic stress disorder checklist (PCL5). All 155 women (100%) had type 1 genital mutilation and six women (3.9%) had PTSD. The mean (SD) score of the PTSD symptoms was 27.73 (6.79), ranging from 0 to 80. The PTSD symptoms enhanced by increasing age at FGM/C and decreasing socio-economic levels.

## Background

Female Genital Mutilation/ Cutting (FGM/C) refers to any procedure involving intentionally cutting, injuring, or removing female genitalia [1]. The World Health Organization (WHO) classifies FGM/C into four distinct categories. Type I involves the partial or total removal of the clitoral glans, and/or the prepuce/ clitoral hood. Type II refers to the partial or complete removal of the clitoral glans and the labia minora, with or without removal of the labia majora. Infibulation or type III is defined as narrowing of the vaginal opening by sealing with incision and reposition of the labia minora and labia majora with or without clitoral incision. Type IV is known as the unclassified type and includes other harmful measures that are applied to the external genitalia of women, such as piercing, cauterizing, splitting or tearing the clitoris [2].

The United Nations Children's Fund (UNICEF) estimates that at least 200 million women and girls alive today living in 30 countries have undergone FGM/C. Although FGM/C is declining, over 4 million girls are at risk of circumcision yearly worldwide [3]. FGM/C is prevalent in the south and west regions of Iran, often in rural areas and in the suburbs of Hormozgan, Kermanshah, Kurdistan, and West Azerbaijan provinces, varying from 40 to 85% [4].

Removing and damaging healthy and normal female genital tissue interferes with the natural function of body and results in the short- and long-term health consequences [5]. The results of some studies demonstrated that women with genital mutilation experienced more anxiety, fear, and the symptoms of a panic attack and have less self-confidence compared to the women without genital mutilation [6, 7]. However, some other studies reported no correlation between FGM/C and mental disorders [8–10]. In some societies, FGM/C triggers PTSD [5, 11, 12].

Post-traumatic stress disorder is a disorder resulting from an abnormal response to a traumatic event. The person experiences the event frequently through disturbing thoughts or nightmares. These memories come to the individual’s mind suddenly or occur when the individual is in a similar situation. The symptoms of PTSD include re-experiencing the event in the mind, making negative alterations in mood and cognition, and reactions, such as restlessness, difficulty concentrating and sleeping, anger, etc. [13].

In a systematic review conducted in 2019, among 16 studies conducted on the psychological consequences of FGM/C, only nine studies examined PTSD and its prevalence varied from 17.5 to 30.4%. However, only one of these nine studies was of acceptable quality for review and most of the studies did not based on FGM/C type. Therefore, it is suggested to conduct more rigorous studies on the psychological effects of FGM/C and its relationship with culture in different societies [14]. In another review study, it is recommended to use standard tools in future studies, due to the contradictory results of studies about the psychological consequences of FGM/C [6].

Raising awareness on the consequences of FGM/C is essential for its prevention. Given that the type and prevalence of FGM/C in different societies affect its consequences and considering that no study has been done in Iran on PTSD following FGM/C, the present study aimed to assess the prevalence and severity of PTSD and its associated factors among Iranian women with genital mutilation.

## Method

### Study design and participants

This cross-sectional study was conducted on 155 women with FGM/C referred to the health centers of Mahabad, located in west of Iran, from October 2020 to April 2021. The inclusion criteria were women with FGM/C aged 18–45 years. The exclusion criteria included current pregnancy and breastfeeding, having any known physical or mental disease, and history of traumatic events during the last six months.

### Sample size

The sample size was calculated 155 subjects using the formula $\mathrm{n}=\frac{{\mathrm{Z}}_{1-\frac{\mathrm{\alpha }}{2}}^{2} {\delta }^{2}}{{\mathrm{d}}^{2}}$ and based on the PTSD variable in the study of Bahari et al. [15] and considering SD = 7.1, d = 0.05 around the mean = 22.3, α = 0.05 with 95% confidence coefficient.

### Sampling method

The sampling was done using the convenience sampling method. The first researcher attended the health centers, extracted the telephone numbers of the women from the health records, checked the inclusion and exclusion criteria during the telephone call, and invited the eligible women to participate in the study. The women willing to participate in the study were invited to attend health centers to complete the written informed consent form. Then, the first researcher (a female midwife) in the presence of a female gynecologist determined the type of genital mutilation, according to WHO classification [2]. Women’s height and weight were measured and body mass index (BMI) was calculated as weight (kg)/ height^2^(m) [16].

### Data collection tools

The demographic and obstetric characteristics included the variables of age, duration of marriage, number of children, level of education, occupation, family income, type of residence, type of delivery, etc.

### PTSD checklist for DSM-5 (PCL5)

The symptoms of PTSD were assessed using PCL5, developed in 2013. PCL5 consists of four domains, including criterion B (re-experiencing symptoms), criterion C (avoidance of stimuli), criterion E (negative alterations in mood or cognition), and criterion D (arousal or reactivity). PCL5 includes 20 items, rating from 0 to 4 and total score range is between 0 and 80. The cutoff point of 33 is important for clinical diagnosis [17]. The Cronbach’s alpha coefficient of this instrument was reported as 0.91–0.94 and the test–retest reliability of the instrument was obtained 0.82 [18]. Cronbach’s α and the test–retest results for the Persian version of the questionnaire were more than 0.70 [19].

### Data analysis

The data were analyzed using SPSS/21 software. The Kolmogorov–Smirnov test was used to measure the normality of data distribution. The independent t-test, Pearson correlation coefficient, and ANOVA were applied for data analysis. In order to control the confounding variables, the independent variables with P- value lower than 0.2 in the bivariate test were entered into the multivariate linear regression model using Backward strategy.

## Results

### Sociodemographic characteristics

All 155 married women (100%) had type 1 genital mutilation. The mean (SD) age of participants was 31.46 (6.94) year. The minimum age at FGM/C was two years and the maximum age was ten years. Most of the participants (78.7%) had undergone FGM when they were aged 4 to 8 years old. The educational level of most of the participants (49.7%) was high school and diploma and most of them (77.4%) were housewives. The majority of women (61.9%) reported their family income as inadequate. There was no significant difference in the mean score of BMI between the two groups (Table 1).

**Table 1:** The demographic and obstetric characteristics of the participants and its relationship with PTSD, 2021 (n = 155)

Variables	N (%)	PTSD Mean (SD)	p-value
Age^*^ (year)	31.46 (6.94)	27.73 (6.79)	r = 0.026, p = 0.501^a^
Mutilation age* (year)	5.78 (1.58)	27.73 (6.79)	r = 0.130, p = 0.016^a^
Duration of marriage* (year)	9.33 (5.68)	27.73 (6.79)	r = 0.013, p = 0.940^a^
Number of children*	1.83 (0. 9)	27.73 (6.79)	r = 0.025, p = 0.215^a^
BMI^*^	25.98 (1.88)	27.73 (6.79)	r = − 0.100, p = 0.038^a^
Occupation			f = 1.531, p = 0.256^b^
Housekeeper	120 (77.4)	28.71 (6.71)	
Working outdoors	21 (13.5)	26.95 (6.84)	
Working at home	14 (9)	27.80 (7.23)	
Level of education			f = 1.824, p = 0.015^b^
Elementary/middle school	16 (10.3)	29.02 (7.42)	
High school/Diploma	77 (49.7)	28.13 (8.05)	
College education	62 (40)	25.23 (7.80)	
Family income level			f = 0.208, p = 0.924^b^
Inadequate (under $300 per month)	96 (61.9)	30.11 (10.21)	
Partially adequate (between $300–500 per month)	56 (36.1)	26.74 (9.91)	
Adequate (over $500 per month)	3 (1.9)	20 (8.18)	
Birth type			t = − 0.367, p = 0.771^c^
NVD	86 (55.5)	27.80 (13.21)	
C/S	46 (29.7)	27.66 (11.53)	
Type of residence			f = 1.730, p = 0.002^b^
Private	42 (27.1)	24 (9.19)	
Leased	79 (51)	28.16 (10.45)	
Father-in-law’s house	34 (21.9)	30. 02 (9.44)	

^a^Pearson Correlation, ^b^ANOVA, ^c^Independent t test

### Type of FGM and magnitude of PTSD

In this study, six women (3.9%) had PTSD. The mean (SD) score of the PTSD symptoms among the women was 27.73 (6.79) in the attainable score of 0–80. The mean (SD) of re-experiencing symptoms was 6.12 (2.7) in the obtainable domain score of 0–20. The mean (SD) of the avoidance of stimuli was 4.78 (2.24) in the achievable domain score of 0 to 8. The mean (SD) of the negative alterations in mood or cognition was 9.38 (3.38) in the attainable domain score of 0–28. Further, the mean (SD) of the arousal domain was 7.45 (2.62) in the obtainable domain score of 0 to 24. Then, the average standard score of the different domains was estimated based on the following formula:

$${\text{Standard}}\;{\text{score}}\;{\text{in}}\;{\text{dimensions}} = \frac{{({\text{Total}}\;{\text{score}}\;{\text{of}}\;{\text{each}}\;{\text{dimension}} - {\text{Minimum}}\;{\text{score}}\;{\text{of}}\;{\text{that}}\;{\text{dimension}})}}{{({\text{Maximum}}\;{\text{score}}\;{\text{of}}\;{\text{each}}\;{\text{dimension}} - {\text{Minimum}}\;{\text{score}}\;{\text{of}}\;{\text{that}}\;{\text{dimension}})}} \times 100.$$

Accordingly, the lowest complaint was related to the re-experiencing symptoms with a modified mean of 30.6 and the highest complaint was associated with the avoidance of stimuli with a modified mean of 59.75 (Table 2).

**Table 2:** Mean (SD) of the PTSD and its domains, 2021 (n = 155)

Variable	Mean (SD)	Obtainable score	Adjusted Mean (0–100)
Re-experiencing	6.12 (2.7)	0_20	30.6
Avoidance	4.78 (2.24)	0_8	59.75
Negative mood	9.38 (3.38)	0_28	33.5
Arousal	7.45 (2.62)	0_24	31.04
Total PTSD score	27.73 (6.79)	0_80	34.66

### Factors associated with PTSD

The bivariate tests showed a significant relationship between PTSD score and mutilation age (P = 0.016), body mass index (P = 0.038), education (P = 0.015), and type of residence (P = 0.002) (Table 1). After entering variables with p < 0.2 in the multivariate linear regression model, the predictors of PTSD severity included age at FGM/C, education, and type of residence, which generally explained 48.1% of the variance (Table 3).

**Table 3:** Predictors of PTSD in women with FGM/C, 2021 (n = 155)

Variable	β (CI 95%)*	P^#^
Age mutilation	1.95(0.009to 1.461)	0.043
Education (Reference: MSc)		
Elementary/middle school	1.43(1.445to 8.946)	0.015
High school/ Diploma	1.28(1.741to 8.165)	0.020
Associate Degree/ Bachelor	1.23(-1.907to 8.224)	0.220
Type of residence (Reference: private)		
Leased	0.60(2.046to 3.806)	0.013
Father-in-law’s house	1.41(0.997to 5.938)	0.036
Adjusted R2	48.1	

^*^Confidence interval 95%; ^#^Multivariate linear regression

## Discussion

The present study aimed to assess the prevalence and severity of PTSD and its associated factors among Iranian women with genital mutilation.

### Type of FGM and magnitude of PTSD

The results of the present study indicated that six women (3.9%) had PTSD. The mental health status of 66 circumcised women migrating from Africa to the Netherlands was investigated in a study conducted in 2012 and the results indicated that about 54% of participants had type III FGM, 14% type II and 32% type I. Further, approximately, 16% of circumcised women had the symptoms of PTSD [20]. In another study, 17.5% of African women with genital mutilation had PTSD symptoms [21]. The results of a study performed in Egypt indicated that 19% of women with genital mutilation exhibited symptoms of PTSD [22]. Further, the prevalence of PTSD among circumcised women was reported to be 30.4% in a study conducted in Senegal [23]. In most of studies, the prevalence of PTSD was not reported by the type of FGM/C and only a few studies stratified the results based on FGM/C type [5, 21, 23]. The prevalence of PTSD in the present study is much lower than the prevalence reported in various studies with the same sampling method, which may be due to the high prevalence of type I of FGM/C in the study area. On the other hand, the acceptance of FGM/C among the study population may be acted as a protective factor against PTSD. Early studies also reported that the prevalence of psychological trauma and PTSD increases in societies with low prevalence of FGM/C and its social rejection [14, 22].

In the present study, the mean (SD) score of PTSD symptoms among Kurdish women with type 1 of FGM/C was 27.73 (6.79). In a study performed in north of Iraq [24], sharing borders with Iran, the mean (SD) score of PTSD among circumcised Kurdish girls aged 8–14 years was 44.3 (13.73) and the severity of symptom was very high compared to the findings of the present study, which may be attributed to the low prevalence of FGM/C in the north of Iraq, differences in the age groups, and not stratifying the results based on FGM/C type. The study population in the aforementioned study was single girls with a mean age of 12.2 years and in the present study was married women with a mean age of 31.46 years. Further, in a study conducted in Kenya [25], the mean score of PTSD symptoms among women with genital mutilation migrating from Somalia was 41.16 (16.1), as these refugee women had experienced many difficulties and challenges before, during, and after migration, they were more likely to experience higher levels of PTSD. In the present study, the severity of PTSD among the participants were relatively low, which may be due to convenience sampling of the study, the limited injury in genitalia and the social acceptance of the practice.

### Associated factors of PTSD

In the present study, the age at FGM/C was regarded as the predictor of PTSD among the women. Namely, individuals who were older at the time of genital mutilation exhibit more PTSD symptoms. Consistent with the findings of the present study, the results of a study conducted on Somali women revealed a positive relationship between the age at FGM/C and PTSD symptoms [25]. Vloeberghs et al. [20] also reported that the severity of PTSD symptoms was higher among women who were older at the time of genital mutilation.

In addition, the level of education and type of residence were considered as other predictors of PTSD in the present study, so that the severity of PTSD symptoms increased among women with lower level of education and those who live in the house of the father-in-law. In fact, circumcised women with low social and economic status demonstrated more PTSD symptoms. Knipscheer et al. [21] reported that the low economic status was significantly associated with psychological trauma among circumcised girls and women, which is in line with the results of the present study. Further, Vloeberghs et al. [20] addressed that full housewife women experienced more anxiety and depression compared to the employed circumcised women.

### Strength and limitation of the study

The strengths of the present study included using standard questionnaire and reporting the results in detail for type 1 FGM. Moreover, the findings of the study provided evidence for planning a future advanced study. The main limitation of study is the use of the convenience sampling method. Also, the results of the present study cannot be generalized to other contexts or cultures, since it was conducted in one geographical area with specific ethnicity.

## Conclusion and recommendation

In the present study, the prevalence and severity of PTSD among the participants were relatively low, which may be due to the convenience sampling of the study, the limited injury in genitalia, and the social acceptance of the practice. Based on the results, the PTSD symptoms enhanced by increasing age at FGM/C and decreasing socio-economic levels. Conducting similar studies in societies with high prevalence of other types of FGM/C is strongly recommended.

## Data Availability

The data sets used and/or analysed during the current study are available from the corresponding author on reasonable request.

## Abbreviations

FGM/C: Female genital mutilation/cutting
PTSD: Post-traumatic stress disorder
DSM-5: Diagnostic and statistical manual of mental disorders-the fifth version
PCL5: Post-traumatic stress disorder checklist for DSM-5
WHO: World Health Organization
UNICEF: United Nations Children's Fund
CI: Confidence interval
SD: Standard deviation
